# High-Resolution Linkage Map and QTL Analyses of Fruit Firmness in Autotetraploid Blueberry

**DOI:** 10.3389/fpls.2020.562171

**Published:** 2020-11-16

**Authors:** Francesco Cappai, Rodrigo R. Amadeu, Juliana Benevenuto, Ryan Cullen, Alexandria Garcia, Adina Grossman, Luís Felipe V. Ferrão, Patricio Munoz

**Affiliations:** ^1^Blueberry Breeding and Genomics Lab, Horticultural Sciences Department, University of Florida, Gainesville, FL, United States; ^2^Forage Breeding and Genetics Lab, Agronomy Department, University of Florida, Gainesville, FL, United States

**Keywords:** Vaccinium, southern highbush blueberry, fruit detachment force, polyploids, machine harvest

## Abstract

Blueberry (*Vaccinium corymbosum* and hybrids) is an autotetraploid crop whose commercial relevance has been growing steadily during the last 20 years. However, the ever-increasing cost of labor for hand-picking blueberry is one main constraint in competitive marketing of the fruit. Machine harvestability is, therefore, a key trait for the blueberry industry. Understanding the genetic architecture of traits related to machine harvestability through Quantitative Trait Loci (QTL) mapping is the first step toward implementation of molecular breeding for faster genetic gains. Despite recent advances in software development for autotetraploid genetic mapping, a high-resolution map is still not available for blueberry. In this study, we crafted a map for autotetraploid low-chill highbush blueberry containing 11,292 SNP markers and a total size of 1,953.97 cM (average density of 5.78 markers/cM). This map was subsequently used to perform QTL analyses in 2-year field trials for a trait crucial to machine harvesting: fruit firmness. Preliminary insights were also sought for single evaluations of firmness retention after cold storage, and fruit detachment force traits. Significant QTL peaks were identified for all the traits and overlapping QTL intervals were detected for firmness across the years. We found low-to-moderate QTL effects explaining the phenotypic variance, which suggest a quantitative nature of these traits. The QTL intervals were further speculated for putative gene repertoire. Altogether, our findings provide the basis for future fine-mapping and molecular breeding efforts for machine harvesting in blueberry.

## Introduction

It is not uncommon to find plants with different ploidy levels within the same genus. In fact, such chromosomal variation are not only tolerated by plant genomes, but can also drive evolution, for example, promoting speciation (Adams and Wendel, 2005). Polyploid organisms are classified as either allopolyploids or autopolyploids, depending on the degree of divergence between their subgenomes (Brubaker et al., 1999). Autopolyploids, such as blueberry (*Vaccinium corymbosum* L.), potato (*Solanum tuberosum* L.), and alfalfa (*Medicago sativa* L.), contain multiple copies of the same chromosome set, which can all pair and exchange genetic material during gamete formation. In contrast, allopolyploids, such as wheat (*Triticum aestivum* L.), coffee (*Coffea arabica* L.), and strawberry (*Fragaria ananassa* Duch.) contain two or more divergent genomes, which show bivalent pairing and have disomic inheritance, like diploid organisms. While in diploid systems the study of allelic inheritance is relatively simple, polysomic inheritance in autopolyploids increases the number of possible genetic configurations and impacts downstream genetic analyses, including linkage map construction and quantitative trait loci (QTL) mapping (Bever and Felber, 1992).

QTL mapping is the association study between phenotypic and genetic variants usually performed by tracking recombination events of the possible QTL along chromosomes (Broman and Sen, 2009). In breeding programs, QTL analysis has been historically used to understand the genetic basis of complex traits, which can ultimately support the implementation of marker-assisted selection (MAS). To this end, the development of a reliable linkage map and haplotype inference are both considered crucial steps. A classical approach for autopolyploids relies on building individual maps at each homolog level using a combination of diploid software and single-dose markers, *i.e.*, markers that segregate in a diploid fashion (1:1 and 1:2:1) (Mollinari and Garcia, 2019). Although widely adopted, this strategy has a few shortcomings, including the use of only a subset of markers, the low-power to detect markers in repulsion, and the use of *ad-hoc* procedures to assemble the Linkage Groups (LGs) into homology groups. Just recently, new methods addressing autotetraploid genetics and higher orders of segregation patterns have been reported. Software such as TetraploidSNPmap (Hackett et al., 2017), an updated version of the TetraploidMap (Hackett et al., 2007), polymapR (Bourke et al., 2018), and mappoly (Mollinari and Garcia, 2019) have overcome those limitations and allowed the construction of high-density linkage maps using SNP data and multi-dose markers. Potato (Da Silva et al., 2017), sweetpotato (*Ipomoea batatas* L.) (Mollinari et al., 2019) and forage grasses (Ferreira et al., 2019; Deo et al., 2020) are examples of autopolyploid crops that have benefited from the utilization of such modern methodologies in QTL inference and linkage mapping analyses. However, to the best of our knowledge, no high-density linkage map has been built for autotetraploid blueberries. Linkage maps reported for blueberry have relied on diploid populations (Rowland et al., 2014) and/or used a small number of markers and individuals (McCallum et al., 2016; Schlautman et al., 2018). A high-density linkage map has only been built for cranberry (*Vaccinium macrocarpon* Ait.) (Schlautman et al., 2017), a diploid-relative of blueberry.

Highbush blueberry is an autotetraploid crop (2n = 4x = 48), considered the second most important soft fruit, after strawberry (Brazelton et al., 2017). Despite the considerable dedicated world acreage and varietal improvement through traditional breeding, blueberry still remains one of the most expensive commonly sold fruits by weight (USDA Economic Research Service, 2020). A main factor contributing to these sustained prices is the high production cost, mostly due to the laborious hand-picking process, which can represent more than 50% of the total production cost (Ehlenfeldt and Martin, 2002; Olmstead and Finn, 2014; Gallardo et al., 2018). In addition, a number of countries, such as the USA, are experiencing labor shortages, further exacerbating this issue (Roka and Guan, 2018). A possible solution that has been tested in blueberry fields is the mechanization of the harvesting operations. Mechanical harvesting of berries can lead to a considerable (up to 80%) reduction in cost, while also reducing food-borne illnesses caused by improper produce handling by laborers (Berger et al., 2010; Olmstead and Finn, 2014). Machine harvesting consists of metal rods shaking the blueberry bushes, forcing berries to fall. To successfully implement machine harvest, the following traits, alongside with plant architecture, are crucial: (I) high berry firmness to withstand physical damage; (II) high detachability in ripe (blue) fruit; and (III) low detachability in unripe (green) fruit.

Firmness is a genetically controlled trait that has long been among the focus areas of blueberry breeding programs (Edwards et al., 1974; Cappai et al., 2018; Ferrão et al., 2018). It is also a desirable trait across the whole market chain: for growers, less fruit is discarded due to physical damage; for retailers, firmer berries have a longer post-harvest and shelf life; and for consumers, firm texture positively correlates with taste preferences (Mehra et al., 2013; Olmstead and Finn, 2014; Yu et al., 2014; Gilbert et al., 2015). At a molecular level, fruit firmness is intertwined with fruit ripening, and it is the result of a complicated network of interactions between two main hormones, ethylene and abscisic acid (ABA), and the cell wall disassembly process (Brummell, 2006; Vicente et al., 2007; Chiabrando and Giacalone, 2011; Cappai et al., 2018). The exact cascade of events that leads to fruit softening has not been completely elucidated in blueberry, even though current evidence indicates that pectin solubilization in the cell wall could be one of the main outcomes (Lara et al., 2004; Vicente et al., 2007; Angeletti et al., 2010; Beaudry et al., 2016). Fruit Detachment Force (FDF), or abscission force, is the force required for a single berry to detach from a stem at the point of the pedicel junction. Machine harvesting requires a large differential between the FDF of green and ripe berries in order to maximize the number of ripe berries that detach from the plant at a defined force point (Malladi and NeSmith, 2010; Malladi et al., 2012; Vashisth et al., 2015). In addition, the force required to detach ripe berries should also be low in absolute terms to ensure efficient fruit removal while avoiding excessive damage to the plant itself. Molecular mechanisms underlying fruit abscission have been extensively studied in tomato (*Solanum lycopersicum* L.) and *Arabidopsis*, which point to the role of MADS-box family transcription factors on the regulation of the pedicel-abscission zone development (Ferrándiz, 2002; Nakano et al., 2012; Ito and Nakano, 2015). Cell wall-modifying proteins and programmed cell death at the abscission zone have been shown to play a role in the cell separation processes (Roberts et al., 2002; Tsuchiya et al., 2015). There is also strong evidence for the interplay between phytohormones in regulating fruit abscission, with ethylene and auxin enhancing and inhibiting the process, respectively (Estornell et al., 2013; Patterson et al., 2016). In blueberry, comparison of two genotypes contrasting in FDF levels provided preliminary insights into differential expression of genes related to phytohormone pathways (Vashisth et al., 2015).

Here, we aimed to develop a high-resolution linkage map for autotetraploid blueberry and perform QTL mapping to reveal the genetic architecture of the firmness trait. In addition, exploratory QTL analyses were performed for firmness retention and FDF traits considering single evaluations. To this end, we phenotyped a large mapping population for berry firmness and FDF, and genotyped it using a high marker density. Genomic regions associated with the phenotypes were reviewed for their gene repertoire. Overall, our findings provide the basis for future MAS implementation and fine mapping to identify causal variants and elucidate the molecular basis underlying fruit firmness.

## Materials and Methods

### Plant Growth Conditions

The population used in this study is an outcrossing F1 full-sib population derived from heterozygous parents. It consisted of 237 individuals planted in 2010 derived from a cross between two varieties, “Sweetcrisp” and “Indigocrisp,” both released by the Blueberry Breeding Program at the University of Florida (Lyrene, 2009, 2016). The population was maintained in Waldo, Florida (29°24'30.2“N 82°08'32.6”W) on a private commercial farm. Plant spacing was ~86 cm within rows and 2 m between rows. Following the farm's commercial practices, plants received 1.5 gallons of water per plant a day injected with 180 ppm sulfuric acid through a drip irrigation system to adjust soil pH. During the growing season (February through October), a 10-5-5 liquid fertilizer was also applied through the irrigation system. Plants were pruned in the summer months of June and July. Insecticide for spotted wing drosophila (*Drosophila suzukii* Matsumura) as well as fungicides (Pristine, Switch, and Proline) were applied six times a year to manage diseases and crop damage. In winter months (December and January), freeze protection measures were applied as necessary.

### Plant Genotyping

Genomic DNA was extracted in 2018 from leaf tissue of each individual in the mapping population and both parental cultivars using a 2% CTAB extraction method (Xin and Chen, 2012). Library preparation and sequencing were performed by RAPiD Genomics (Gainesville, FL, USA) using a sequence capture approach. Briefly, 6,000 custom-designed biotinylated probes of 120-mer were selected based on the distribution on the “Draper” reference genome (Benevenuto et al., 2019), and previous testing on the parental cultivars. Sequencing of the entire population was carried out in the Illumina HiSeq platform using 150 cycle paired-end runs. Raw reads were demultiplexed and barcodes were removed. Data was cleaned and trimmed at the 3′ end by removing bases with quality scores lower than 20 and reads with more than 10% of the bases with quality scores lower than 20, using Fastx Toolkit v.0.0.14 (http://hannonlab.cshl.edu/fastx_toolkit/). Trimmed reads were aligned to the “Draper” blueberry reference genome using MOSAIK v. 2.2.30 (Lee et al., 2014). The 12 largest chromosomes of each homeologous set from the “Draper” genome were used as reference (Colle et al., 2019). SNPs were called using FreeBayes v.1.0.1 (Garrison and Marth, 2012), targeting the 6,000 probe regions. SNPs were further filtered by (I) minimum mapping quality of 30; (II) mean depth of coverage of 40; (III) maximum missing data of 50%; IV) minor allele frequency of 0.01; (V) only biallelic loci; and (VI) no monomorphism. A total of 21,513 SNPs were kept, after these filtering steps. Subsequently, read counts per allele and individual were extracted from the variant call file using vcftools v.0.1.16 (Danecek et al., 2011). The updog R package was used to call the tetraploid allele dosages based on the read counts and “f1” model (Gerard et al., 2018). The “f1” model implemented in the software uses parental information as a baseline for estimating the allele dosages of the progenies. The posterior probability means per SNP for each individual were rounded toward the closest integer and used as our genotypes.

### Plant Phenotyping

Mature fruits were collected at peak ripeness, in March and early April of 2018 and 2019. Berries were stored at 4°C overnight, brought to room temperature, and their firmness was measured using the FirmtechTM 2 firmness tester (BioWorks, Wamego, KS). Berries were placed on the machine with their equatorial axis perpendicular to the instrument's surface and probe. The force-deformation response of compressed berries was measured in grams per 1 mm of deflection (g/mm) (Ehlenfeldt and Martin, 2002). A sample size of 25 berries per genotype was used in 2018 and a sample size of 50 berries per genotype was used in 2019, as this sample size was reported to increase accuracy (Cappai et al., 2018). Berry firmness measurements were averaged per genotype within each year.

Fruit firmness retention and fruit detachment force traits were measured only in the 2019 year following input by growers and other stakeholders. For firmness retention, berries previously used to measure firmness in 2019 were stored for 4 additional weeks in a climate-controlled chamber at 4°C in the dark. They were then removed from the chamber, brought to room temperature, and firmness was measured as described before. Then, the differential firmness value of before and after cold storage was computed. Fruit detachment force (FDF) was measured for ripe blueberries and unripe green berries using a DS2 Digital Force Gauge (Imada, Northbrook, Ill.) (Olmstead and Finn, 2014). Berries were placed in the prong of the force gauge and pulled at a 90° angle away from the bush. At the time of berry separation at the pedicel-berry junction, the force measurement was recorded in Newtons. If a berry fell from the bush without being pulled, a force measurement of 0.1N was recorded. The sample size for fruit detachment force was 25 green berries and 25 ripe berries for each parent and offspring in the mapping population. The differential fruit detachment force (or ΔFDF) was calculated per genotype as follows: ΔFDF= (average FDF of ripe berries)—(average FDF of green berries).

### Phenotypic Analysis

We computed adjusted means (ls-means) for each genotype based on a linear model, where genotype and year were considered fixed effects; ls-means for each trait were used as our response variables for the subsequent QTL mapping analyses. To compute genomic heritabilities, we used linear mixed models to estimate the variance components using restricted maximum likelihood estimator approach in the asreml-R software (Butler, 2019). For the firmness trait, we fit the following linear mixed model *y* = *Xb* + *Zg* + *e*, where *y* is the response variable; *b* is the fixed effect of year; *g* is the random effect for the genotype nested within the year where *g* ~ *MVN* (0, *G*_*b*_ ⊗ *K*_*g*_), being *G*_*b*_ a 2*x*2 unstructured variance-covariance matrix for the year effect and *K*_*g*_ the genomic relationship matrix; *e* is the residual effect where *e* ~ *MVN* (0, Σ_*b*_ ⊗ *I*_*g*_), being Σ_*b*_ a 2*x*2 unstructured variance-covariance matrix for the residual term associated with the year variation, and *I*_*g*_ an identity matrix. *X* and *Z* are the fixed and random effects, respectively. *K*_*g*_ was estimated by the average of the genomic relationship matrices computed with the identity-by-descent probabilities for every 1 cM with the genotypic probabilities derived from the linkage map. Heritability (*h*^2^) was computed for within each year as $h^{2}=\sigma_{g\left(b\right)}^{2}/\left(\sigma_{g\left(b\right)}^{2}+\sigma_{e\left(b\right)}^{2}\right)$. For FDF, ΔFDF, and firmness retention, *h*^2^ was computed based on a similar model, but with no year effect.

### Map Crafting and Parameters

The linkage map was built based on the method proposed by Mollinari and Garcia (2019) using the mappoly software and following a series of filtering steps as follows. After genotyping, markers in small unassembled contigs were removed (21,513 remaining markers). Marker genotypes with a probability of correct dosage assignment lower than 0.80 were treated as missing data. Markers with higher than 20% of missing information across individuals were also removed (14,820 remaining markers). Finally, markers with Mendelian segregation distortion were removed, considering a chi-square test with Bonferroni correction assuming an alpha level of significance of 0.05 (14,792 remaining markers).

Pairwise recombination fractions for all the possible linkage phases between pairs of markers were estimated. The phasing configuration with the highest logarithm of the odds (LOD) score was used to build a matrix of recombination fractions. Based on the recombination fraction heatmap, we performed clustering analyses with the unweighted pair group method with arithmetic mean (UPGMA) algorithm to assign 12 clusters for our marker data. Each cluster would represent one of the 12 blueberry linkage groups (LG). The UPGMA marker group assignment was compared with the genome mapping results (*i.e.*, on which chromosome the marker mapped, based on the “Draper” genome assembly). Mismatching markers were removed, resulting in 14,598 markers, which were grouped into 12 distinct LGs. Within each LG, the markers were ordered using the “Draper” reference genome. With the recombination fraction heatmap, the overall map order was checked using the multidimensional scaling (MDS) algorithm as proposed by Preedy and Hackett (2016), and also visually inspected (Margarido et al., 2007). Both methodologies showed similar results. Therefore, we kept the mapping, since the MDS order is not powerful enough to solve local inversions (shorter range distance) and would introduce ordering errors (Preedy and Hackett, 2016; Mollinari et al., 2020).

Multipoint recombination fraction and haplotype phasing were estimated using Hidden Markov Models (HMM) (Mollinari and Garcia, 2019). Briefly, the HMM procedure starts the chain with the first 20 markers, estimates all the possible phases, selects the one with the highest likelihood, then, in order, adds one marker at a time, and reevaluates the map likelihood and distance. We chose a combination of parameters for the HMM procedure so that when a new marker is included, it minimizes the chance of inflating the map while keeping the chain open for other possible phasing configurations and maintaining a high phasing accuracy in a feasible computation time. In the mappoly software, we set the following HMM parameters: *start.set* = *20, thres.twopt* = *10, thres.hmm* = *10, extend.tail* = *200, info.tail* = *TRUE, sub.map.size.diff.limit* = *10, phase.number.limit* = *20, reestimate.single.ph.confirguration* = *TRUE, tol* = *10e-3, and tol.final* = *10e-4*. If the recombination fraction heatmap or the MDS graphics showed possible inversions and/or misplacements of a map segment, alternative orders (chromosome rearrangements) were also evaluated with the HMM procedure and finally, the order with the highest likelihood was kept as the final order. Markers that inflated the map were manually removed during the process or removed during the HMM extension. At the end, 11,292 SNP markers were anchored in the map. Map density was evaluated graphically with LinkageMapView (Ouellette et al., 2018).

### QTL Mapping

The QTL mapping was performed with a random effect interval mapping using a simplified approach derived from Pereira et al. (2020). First, within the final linkage map, we computed the conditional probabilities of each individual haplotype in relation to the 36 possible haplotype combinations from an autotetraploid biparental cross for every 1 cM of the map. To this end, we used an HMM procedure adapted from Lander and Green (1987) and estimated in the mappoly software (Mollinari and Garcia, 2019). The QTL mapping was performed for each trait using these haplotype probabilities as our predictor variables in a random-effect interval mapping (RE-IM) adapted for autopolyploids. Considering *n* individuals, the random effects model for one QTL can be written as *y* = μ + *g*_*q*_ + *e*, where *y* is the response variable, μ is the overall mean effect (fixed), *g*_*q*_ is a vector *n* × 1 with the individual random effects for the QTL, *q*, where $g_{q}\sim MVN\left(0,G_{q}\sigma_{q}^{2}\right)$, being *G*_*q*_ the genetic relationship matrix for this given QTL, where $G_{q}=Z_{q}\Pi Z_{q}^{\prime }$, where *Z*_*q*_ is an incidence matrix *n* × 36 containing the genotype conditional probabilities of each QTL, and Π is a 36 × 36 matrix containing the expected proportion of shared alleles by identity-by-descent between every pair of genotype, $\sigma_{q}^{2}$ is the QTL variance, and $e\sim MVN\left(0,I_{n}\sigma_{e}^{2}\right).$ After fitting a given model for a given QTL, the linear score statistic was computed, which provided a *p*-value for the given QTL that was then converted to −log_10_ (*p* − *value*) (LOP value). To determine the significance threshold for each trait, we performed 1,000 rounds of permutations. In each round, the phenotypic observations were shuffled, a RE-IM model was performed, and the highest LOP values were extracted. Based on the LOP values, we computed the 95% quantile of the second and third peak across all permutations (Doerge and Churchill, 1996; Chen and Storey, 2006). For comparison, we also performed the QTL mapping using fixed-effects interval mapping (FE-IM) as proposed by Hackett et al. (2001). The QTL heritability was computed based on the variance estimates as $h_{q}^{2}=\sigma_{q}^{2}/\left(\sigma_{q}^{2}+\sigma_{e}^{2}\right)$. The individuals best linear unbiased predictions (BLUPs) were decomposed in order to estimate each allelic effect and the combination of alleles for each parent. The qtlpoly software was used to perform QTL analysis (Pereira et al., 2020). Support intervals for the QTL location were computed based on LOP 1-drop rule, similar to the standard LOD 1-drop rule (Lander and Botstein, 1989; Li, 2011).

### Functional Annotation of Genes

Intervals previously selected for each QTL peak based on the LOP 1-drop rule were explored for their putative gene repertoire (Lander and Botstein, 1989; Li, 2011). For firmness, measured over the course of 2 years, overlapping QTL intervals were merged and the whole resulting area was further analyzed. Predicted genes at the QTL intervals in the “Draper” genome were extracted and the correspondent predicted protein sequences were retrieved (Colle et al., 2019). Functional annotation was performed using BLASTp (v.2.9.0) search against the eudicots non-redundant database with an e-value cut-off of 10^−5^ (Altschul et al., 1990). Domain and gene ontology terms were annotated using InterProScan v 5.35-74.0 (Quevillon et al., 2005).

## Results

### Genetic Map

**Figure 1:**
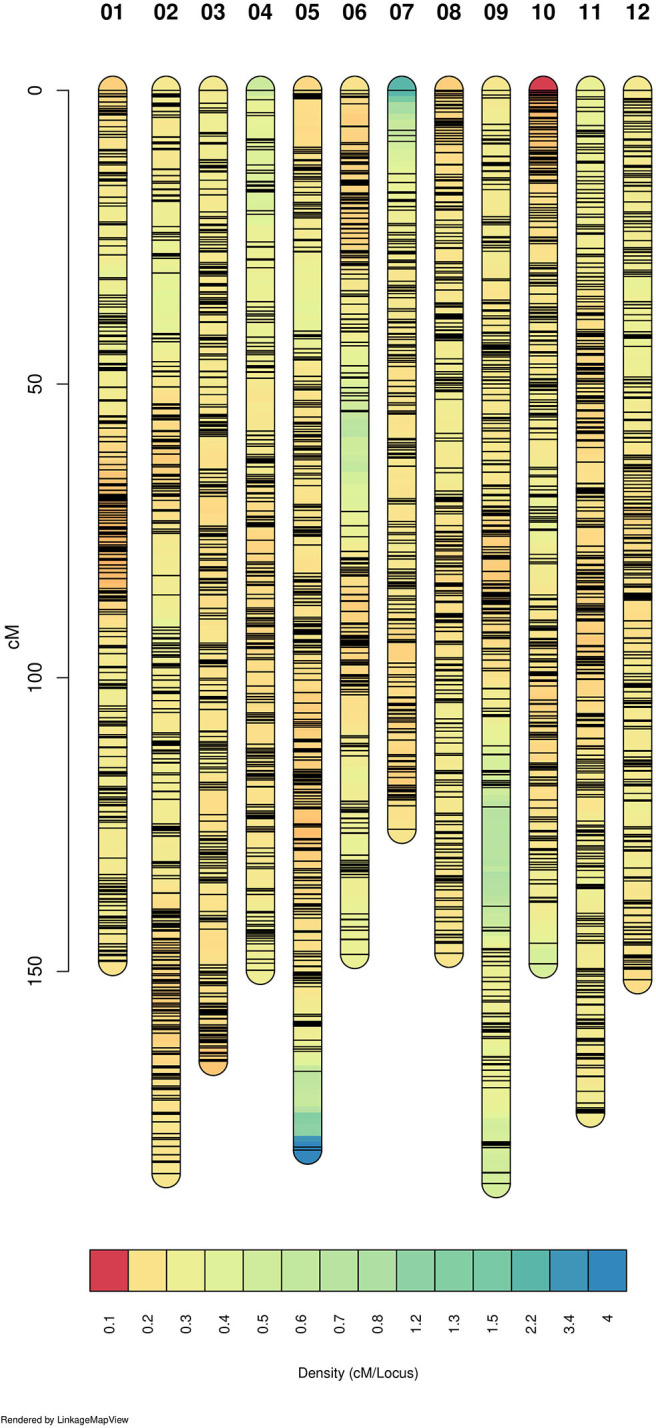
Linkage map density. The 12 linkage groups (LG) are represented and colored according to the marker density. Warmer colors represent denser marker region and each black line shows the marker position. Figure built with LinkageMapView software.

We genotyped 237 individuals and obtained 21,513 SNPs markers. After filtering, we were able to create a tetraploid linkage map that included 11,292 SNP markers with a total size of 1,953.97 cM. This means an average density of 5.78 markers/cM (Table 1). Markers were well-distributed throughout the 12 linkage groups (Figure 1 and Supplementary Figure 1). We found only two gaps between adjacent mapped markers higher than 15 cM (17.01 cM on LG 6 and 16.89 cM on LG 9). The length of each LG ranged from 128.45 cM to 193.78 cM, with an average of 162.83 cM. During map construction, MDS graphical results showed two possible order mismatches between our mapping population and the “Draper” genome. The order mismatches occurred at LG 2 and LG 9 in distal chromosomic segments comparing the reference genome with the suggested MDS order (Supplementary Figure 1). All possible orders and inversions of the individual segments were tested, and the one with the highest likelihood was set as the true order for the map. LGs with their marker order, positions in cM, and parental phasing can be found in Supplementary Table 1.

**Table 1:** Summary statistics of the blueberry genetic map showing the linkage groups (LG), total number of markers assembled (Markers), LG length in centiMorgans (cM), log-likelihood of the LG (LL), and total number of gaps assuming three different thresholds (5, 10, and 15 cM).

				**Total gaps**		
**LG**	**Markers**	**Length**	**LL**	**> 5 cM**	**> 10 cM**	**> 15 cM**
1	932	150.40	−9360.05	1	0	0
2	1,093	189.12	−10724.5	3	1	0
3	1,060	168.28	−10551.79	3	0	0
4	826	153.23	−8961.25	2	0	0
5	1,129	187.46	−11084.64	3	2	0
6	795	154.01	−8094.59	4	1	1
7	749	128.45	−7231.92	2	0	0
8	911	149.05	−10013.13	1	0	0
9	889	193.78	−9367.19	3	1	1
10	955	151.38	−9569.56	2	0	0
11	1,085	175.09	−11101.22	0	0	0
12	868	153.72	−9638.22	1	0	0
**Total**	11,292	1953.97	115698.06	25	5	2

### Heritability and QTL Mapping

**Figure 2:**
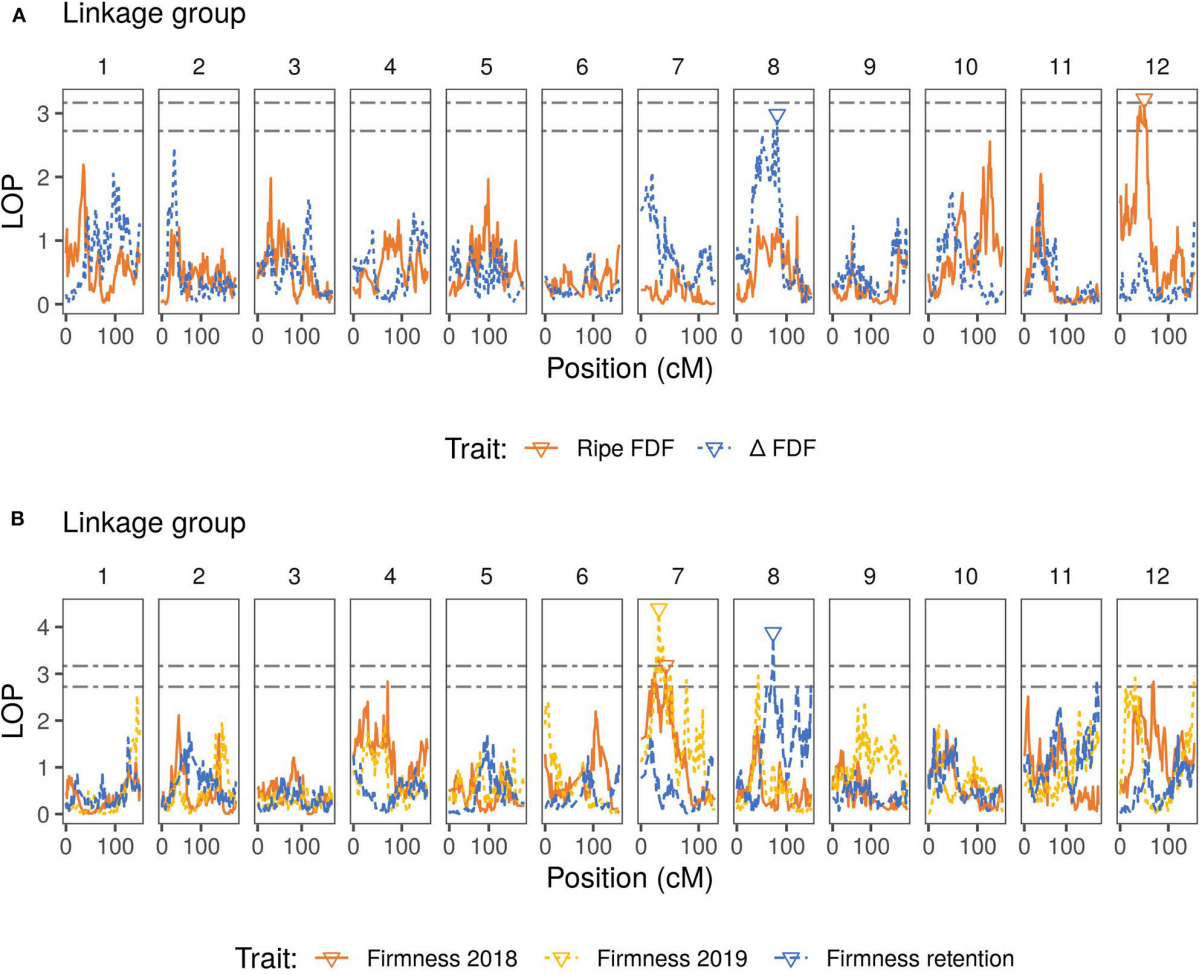
QTL mapping profiles for blueberry machine harvesting traits throughout the 12 linkage groups. LOP is the negative logarithm score of the p-values for the presence of the QTL in the model. Lower triangles indicate inferred QTL positions for each trait. Black dashed lines represent the permutation thresholds considering 95% quantile of the second (top) and third (bottom) highest peak. **(A)** Fruit detachment force (FDF) profiles for ripe fruit and for the difference (△) between ripe and unripe fruit. **(B)** Firmness after one day of storage (for 2018 and 2019 seasons) and firmness retention (difference between 2019 firmness after one day of storage and after four weeks of storage). Firmness retention, Ripe FDF, and △FDF were measured only in year 2019.

Variance estimation for each trait was obtained using linear mixed models. A medium heritability was found for firmness, with similar values observed across the 2 years (Table 2). For the traits measured only in 2019, firmness retention showed the highest heritability (0.75), while blue FDF and delta FDF had a low-medium heritability. For the QTL mapping, the average threshold computed via permutations for the second and third highest peaks had a LOP score of 3.12, and 2.71, respectively. Considering all traits, we mapped a total of five QTLs spanning three different LGs in the blueberry genome. For firmness, we identified one major QTL on LG 7, identified during the year 2018 and 2019, explaining more than 15% of the phenotypic variation (Figure 2 and Table 3). Notably, for firmness retention, a single QTL on LG 8 explained 27% of the phenotypic variance. There was one QTL identified for each Blue FDF and delta FDF in LG 12 and 8, respectively. The QTL mapping LOP profile was similar to the FE-IM model (Supplementary Figure 2).

**Table 2:** Phenotypic averages ($\bar{y}$) for the parents “Sweetcrisp” (P1), “Indigocrisp” (P2), and their offspring (OS), estimated genotypic $s_{G}^{2}$ and error $s_{E}^{2}$ variances, and genotypic heritability *h*^2^ for each trait.

**Trait**	${\bar{\text{y}}}_{\text{P}1}$	${\bar{\text{y}}}_{\text{P}2}$	${\bar{\text{y}}}_{\text{O}\text{S}}$	${\text{s}}_{\text{G}}^{2}$	${\text{s}}_{\text{E}}^{2}$	**h^2^**
Firmness 2018	259.71	238.06	237.7	298.04	356.76	0.46
Firmness 2019	283.26	267.46	271.52	569.29	549.37	0.51
Firmness retention	NA	NA	8.62	563.38	192.14	0.75
Blue FDF	0.43	0.74	0.84	0.02	0.04	0.34
Delta FDF	−0.87	−1.34	1.93	0.11	0.22	0.34

**Table 3:** Summary of significant QTL for each trait including linkage group (LG), highest logarithm of the *p*-value of the QTL (LOP), the peak position in the map in centiMorgans (Peak) with confidence interval assuming 1-LOP drop rule (Lower and Upper bound), SNP position with the maximum LOP score of the peak (Loci), and the QTL heritability (*h*^2^).

**Trait**	**LG**	**LOP**	**Peak**	**Lower**	**Upper**	**Loci**	**h_QTL^2^**
Firmness 2018	7	3.08	43.13	15.06	53.39	12_11134097	0.16
Firmness 2019	7	4.29	31.11	26.42	38.38	12_9191799	0.16
Firmness retention	8	3.78	73.01	69.51	75.47	13_14221092	0.27
Blue FDF	12	3.13	50.04	36.44	55.79	22_10452951	0.19
Delta FDF	8	2.88	81.26	39.11	83.16	13_16002128	0.16

*Firmness retention, Blue FDF, and Delta FDF where measured only in the year 2019*.

### Gene Repertoire at QTL Intervals

The QTL regions using the LOP 1-drop rule spanned genomic intervals from 5.96 to 44.05 cM, encompassing from 1.68 to 9.71 Mb in the reference genome, with each interval harboring hundreds of genes (Supplementary Table 2). These repertoires of predicted genes identified within the QTL intervals were further investigated considering *in silico* functional annotation (Supplementary Table 2). Given the large size of linkage blocks inherent of a typical QTL mapping population, it is difficult at this stage to point out specific candidate genes underlying the variability of the traits. However, a number of biologically plausible genes can be highlighted in a speculative manner. In the QTL interval for firmness-related traits, for example, we highlighted the presence of genes potentially encoding cell-wall modification enzymes (e.g., *endo-1,4-beta-xylanase, beta-galactosidase 3, mannan endo-1,4-beta-mannosidase 7, galacturonosyltransferase-like 1, UDP-glucuronate:xylan alpha-glucuronosyltransferase 2, leucine-rich repeat extensin-like protein*). In the QTL interval for ripe FDF and ΔFDF, we highlighted the presence of putative genes related to MADS-box transcription factors involved in fruit abscission, such as *FUL-like protein/AGL8* and *AGL15*.

## Discussion

Blueberry production costs are subjected to increasingly high hand-harvesting prices. Machine harvesting is an economically viable solution but requires varieties with particular characteristics, such as firm berries and fruits that easily detach only when ripe (Olmstead and Finn, 2014). Despite their importance, the genetic basis underlying the variation of these machine harvest-related traits remains poorly understood in blueberry. Our contributions in this paper are 3-fold: (I) we crafted a high-density genetic map for low chill (*a.k.a.*, southern highbush) autotetraploid blueberry; (II) we mapped potential QTLs associated with machine harvest-related traits, showing their location and effect; and (III) we provided some insights into potential molecular mechanisms for fruit firmness and FDF based on predicted gene functions at significant QTL intervals.

To date, most of the blueberry genetic maps reported in the literature have been constructed using analytical pipelines tailored to diploid organisms, ignoring the fact that allele dosages, polysomic segregation, and multilocus information should be considered in the analyses for a more robust inference in a tetraploid species, such as blueberry. By combining such information with the current genomic resources available, we have created a unifying framework to develop high-quality genetic maps that could be used, for example, as a scaffolding strategy to accomplish future genome assembly projects. Furthermore, the map provides a statement about the inheritance pattern involved in the transmission of genes aiding in the task of haplotype inference. Although other genetic maps were reported in blueberry, high-density polyploid maps are still lacking, while have been already reported for other autopolyploid crop species (Da Silva et al., 2017; Schlautman et al., 2018; Ferreira et al., 2019; Mollinari et al., 2019; Deo et al., 2020). Moreover, given the lack of a high-quality genome assembly, previous blueberry linkage maps were not built considering prior genomic information, and they were based on smaller populations with <100 individuals and lower marker density (Rowland et al., 2014; McCallum et al., 2016; Schlautman et al., 2018). Additionally, two of the maps were based on diploid populations, while the commercial blueberry varieties are autotetraploids (Rowland et al., 2014; Schlautman et al., 2018). While our map shows map inflation, this is lower than has been found in another genetic map built with similar methodology (Mollinari et al., 2020). Three main reasons can be pointed at for this apparent inflation: (I) misplacement of the marker order, (II) genotyping errors, and (III) genotyping dosage errors (Mollinari and Garcia, 2019). Aside from differences in species ploidy level, the genotyping quality and the methodology used for allele dosage calling are also potential reasons to explain the difference in map inflation between these two species.

In this study, we present the highest-density map yet available for tetraploid blueberry (*Vaccinium* spp.), which brings new advances for the blueberry community but also opens questions that warrant further investigation. First, we detected order mismatches between our map and the previously released “Draper” genome (Colle et al., 2019) in LGs 2 and 9. These could be due to real chromosomal translocations specific to these genotypes, genome assembling issues, or mapping artifacts. Another possible investigation is regarding double-reduction landscape in blueberry. Even though there is no strong evidence of its presence (Krebs and Hancock, 1989; Amadeu et al., 2016; McCallum et al., 2016), with such high marker density, double-reduction phenomena could also be investigated extensively in blueberry as it is being done in potato (Hackett et al., 2013; Bourke et al., 2015).

After the genetic map construction, QTL mapping was performed to understand the genetic architecture of firmness, and preliminary insights were sought for other machine harvest related traits. By estimating the number, position, and effect of markers underlying phenotypic variation, QTL results open new opportunities for MAS implementation, which can ultimately accelerate and maximize genetic gains. Despite their potential contribution to breeding programs, phenotype-genotype association studies in blueberry have not been fully utilized. Only recently were the first genome-wide association studies (GWAS) reported for fruit quality and aroma traits (Ferrão et al., 2018; Johnson et al., 2020), while QTL mapping has been performed only for chilling requirement and cold hardiness traits (Rowland et al., 2014).

Using a random-effect model for multiple-QTL mapping, we identified herein one major QTL associated with firmness, which is one of the most important traits for machine harvest. Markers associated with the QTL explained 16% of the phenotypic variance, which suggest firmness is a quantitative train. In accordance with this, previous GWAS analyses in blueberry also detected few and scattered associations between SNPs and fruit firmness using dominant gene action models, which explained a small proportion of the trait variation (Ferrão et al., 2018; Benevenuto et al., 2019). We also detected major QTLs for fruit firmness retention, ripe FDF and ΔFDF traits, providing preliminary insights into the genetic basis of these new traits. Despite the novelty and importance of QTLs for these traits, the stability of the QTLs requires further investigation on multiple years, location and populations. This is because we only quantified these traits for a single season.

Due to the nature of QTL analysis, each significant QTL interval harbored hundreds of genes, making it difficult to point out to specific candidate genes underlying the variability of the traits. Although speculative, the functional annotation of the repertoire of genes within QTL intervals can provide insights for further validation. Overall, all traits considered in this study are tightly related to fruit ripening, senescence, and abscission. These processes are the result of complex interactions of plant hormones, signaling pathways, and transcriptional and cellular modifications that could be playing a role (Seymour et al., 2002; Giovannoni, 2004; Estornell et al., 2013; Cappai et al., 2018). For firmness and firmness retention traits, we highlighted genes related to cell wall modification, such as glycosyltransferases (EC 2.4), glycosylases (EC 3.2) and leucine-rich repeat extensin-like proteins. Fruit softening, both *in vivo* and under storage conditions, has been mainly associated with depolymerization and solubilization of hemicellulose and pectin in the cell wall (Huber, 1983; Brummell, 2006; Chen et al., 2015). For ripe FDF and ΔFDF traits, genes potentially related to the transcriptional regulation of the differentiation and activation of the abscission zone could be interesting candidates, such as FRUITFULL/AGL8 and AGL15. *FRUITFULL (FUL)* is a MADS-BOX transcription factor associated with the differentiation of the dehiscence zone of the silique in *Arabidopsis* (Ferrándiz et al., 2000), and also has significant sequence similarities with the tomato gene *MACROCALYX* (Nakano et al., 2012). The constitutive expression of *FRUITFULL* has been shown to be sufficient to prevent formation of the dehiscence zone (Ferrándiz et al., 2000). *AGAMOUS like 15* (*AGL15*) is also a MADS-BOX transcription factor that maintains a non-senescent state of plant tissues, and whose constitutive expression resulted in delayed floral organ abscission in *Arabidopsis* (Fernandez et al., 2000; Estornell et al., 2013). Other transcription factors detected at the ΔFDF QTL region could also have an indirect role in abscission, such as *OFP7* (Wang et al., 2013)*, SPATULA* (Heisler et al., 2001; Girin et al., 2011), *TCP13* (Koyama et al., 2007), and HHO5 (Butenko and Simon, 2015; Moreau et al., 2016).

Several genes involved in phytohormone pathways were detected at the QTL intervals for all traits. For firmness and firmness retention traits, ethylene and ABA are well-known for their role as ripening promoters and, subsequently, senescence and softening (Suzuki et al., 1997; Zhu et al., 2012; Sun et al., 2013; Cappai et al., 2018). Within the QTL interval for firmness, for example, there were two putative “ethylene-responsive transcription factor (ERFs)” encoding genes. Fine QTL mapping in tomato also detected an ERF gene underlying firmness variation and its increased expression was associated with soft fruit texture in the tomato mapping population (Chapman et al., 2012). Genes related to phytohormone pathways could also be involved in fruit abscission and, therefore, underlying FDF and ΔFDF traits (Lipe and Morgan, 1972; Riov et al., 1990; Chauvaux et al., 1997; Estornell et al., 2013). Despite the copious literature supporting the role of hormones in fruit softening and dehiscence, their pathways and effects are extremely complex and interconnected with other hormones, so many candidates can be speculated to have a role. In addition to hormone-related genes, a diverse set of calcium-related genes were also found in the QTL regions, including some with potential binding, transport, and calcium-activated signal transduction functions. Calcium also has well-documented roles in signaling, water relations, and cell wall modification during fruit ripening in various fruit crops; therefore, these genes may also be underlying the variation of the traits (Conway and Sams, 1987; García et al., 1996; Pilar Hernandez et al., 2006; Ciccarese et al., 2013; Beaudry et al., 2016; Munir et al., 2016; Gao et al., 2019).

Altogether, we reported a high-quality linkage map and candidate QTL regions for firmness and speculatively also for three machine harvest-related traits in autotetraploid blueberry. We used state-of-the-art algorithms for the linkage analysis applied to polyploids and, therefore, our results can be relevant for the polyploid community. We detected QTL associated with machine harvesting traits and characterized their genetic architecture and the potential for MAS implementation in the breeding program. Finally, we reported the repertoire of genes within the QTL intervals. Future efforts to identify causal genes and variants can include a combination of fine mapping, transcriptomics, and functional testing of the genes, such as CRISPR-Cas9 inactivation.

## Data Availability Statement

All datasets generated for this study are included in the article/Supplementary Material. Supplementary Table 1 has the linkage map and inferred parental haplotypes.

## Author Contributions

PM and FC conceived and designed the study. FC, RC, AGa, and AGr conducted the field experiment, phenotyping, and DNA extraction. RA and LF performed statistical genetics analyses. FC and JB performed the SNP filtering and gene annotation. FC and RA wrote the manuscript. All authors read, reviewed, and approved the final version of this manuscript.

## Conflict of Interest

The authors declare that the research was conducted in the absence of any commercial or financial relationships that could be construed as a potential conflict of interest.

## Abbreviations

FDF: Fruit Detachment Force
LG: Linkage Group
GWAS: Genome-Wide Association Study
HMMs: Hidden Markov Models
MDS: Multidimensional Scaling algorithm
QTL: Quantitative Trait Loci
SNP: single nucleotide polymorphism
LOD: Logarithm of the Odds
UPGMA: Unweighted Pair Group Method with Arithmetic mean
MAS: Marker-Assisted Selection
ABA: abscisic acid.
