# Supplementary material for: High-Resolution Linkage Map and QTL Analyses of Fruit Firmness in Autotetraploid Blueberry
Source: Front Plant Sci. 2020 Nov 16;11:562171. doi: 10.3389/fpls.2020.562171 (PMC7701094; doi:10.3389/fpls.2020.562171)
Supplement: Supplementary file 3 [file Table_3.DOCX]

# Supplementary Material

## Supplementary Figure 1


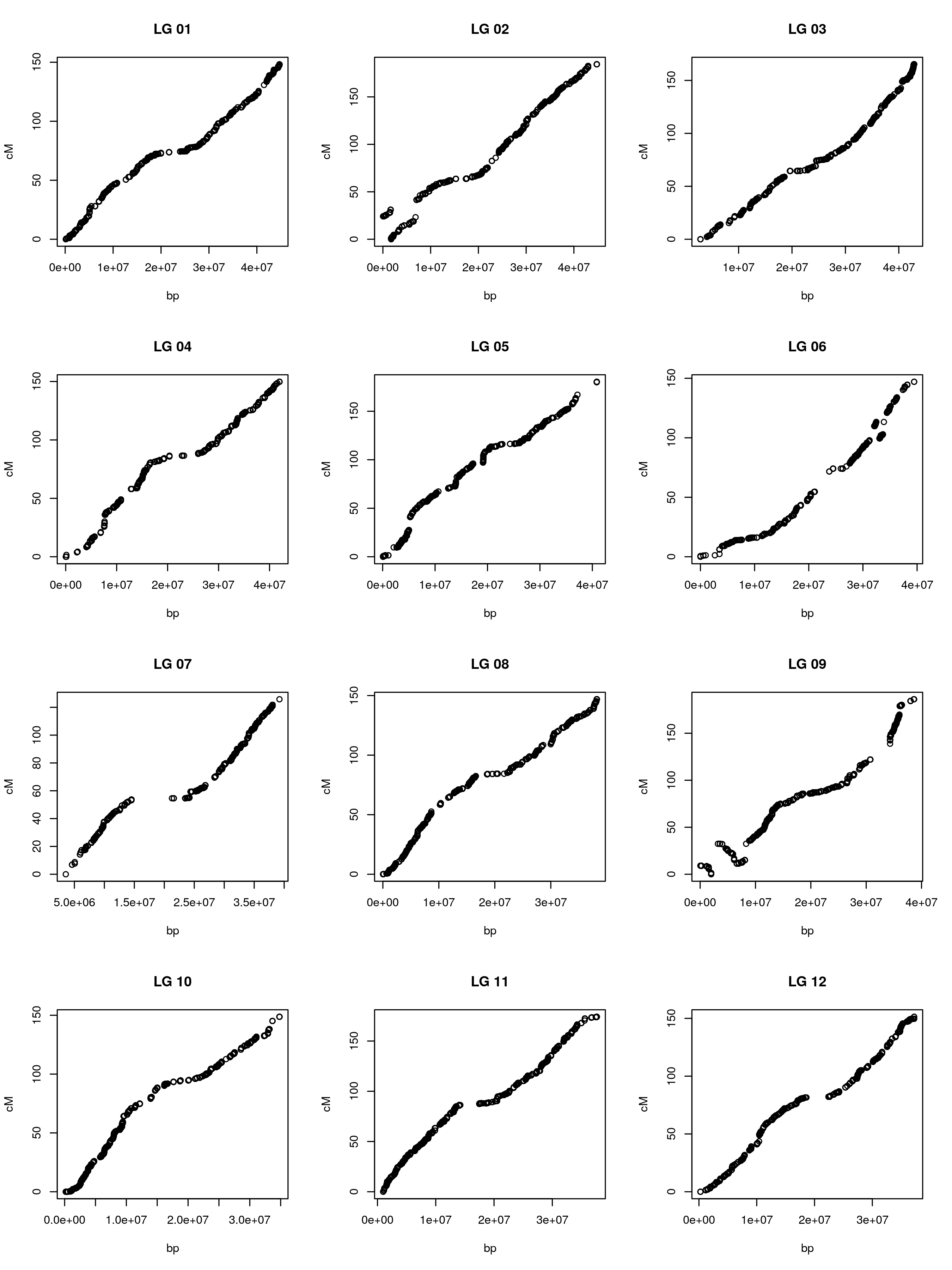


Supplementary Figure 1: Genetic map (y-axis) against the reference genome order (x-axis) for each linkage group (LG). Two possible rearrangements are shown in the distal part of LG2 and LG9. The mapping markers cover the total genomic length. Plateaus are possibly centromeric regions.

Supplementary Figure 2


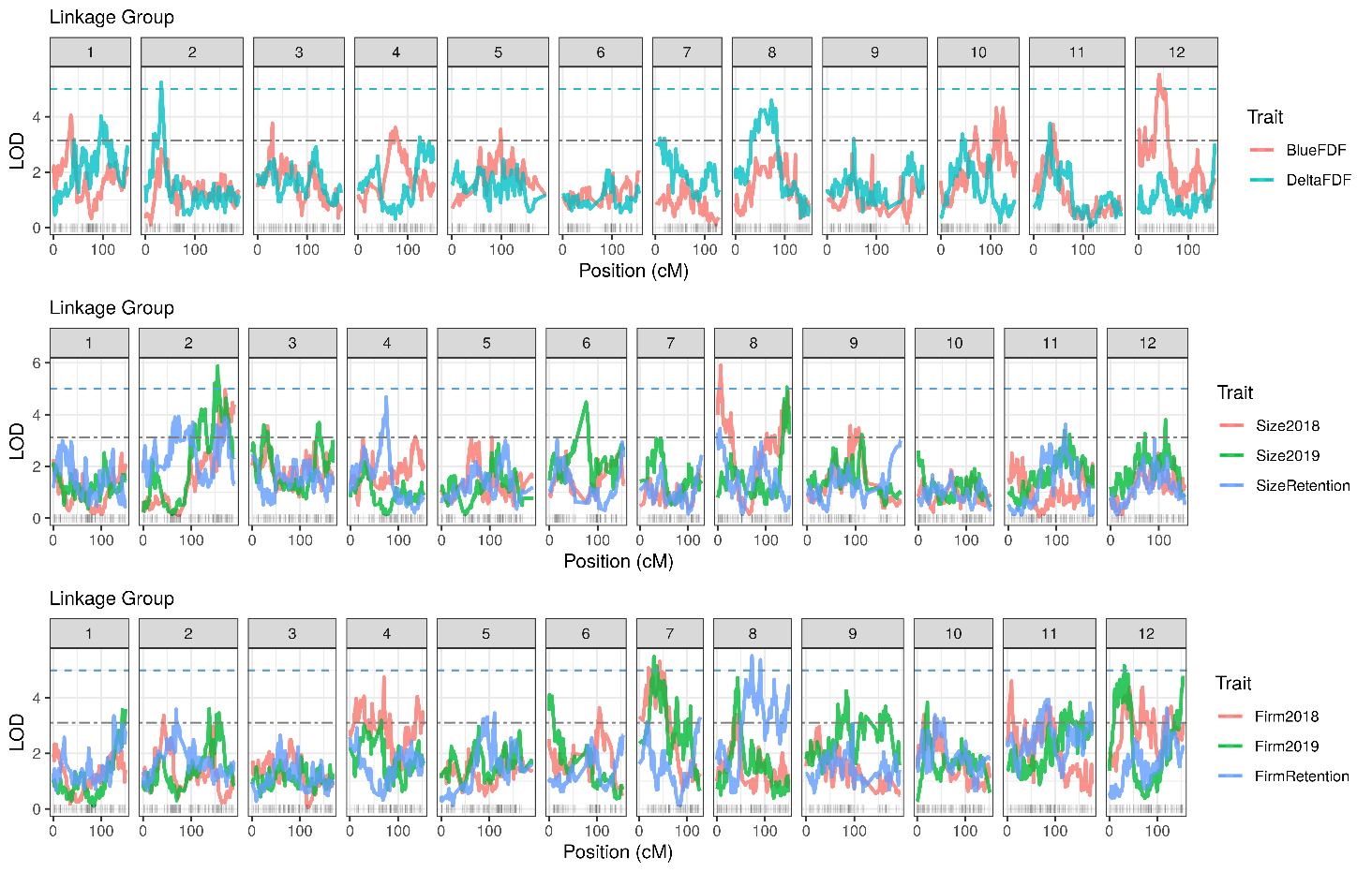


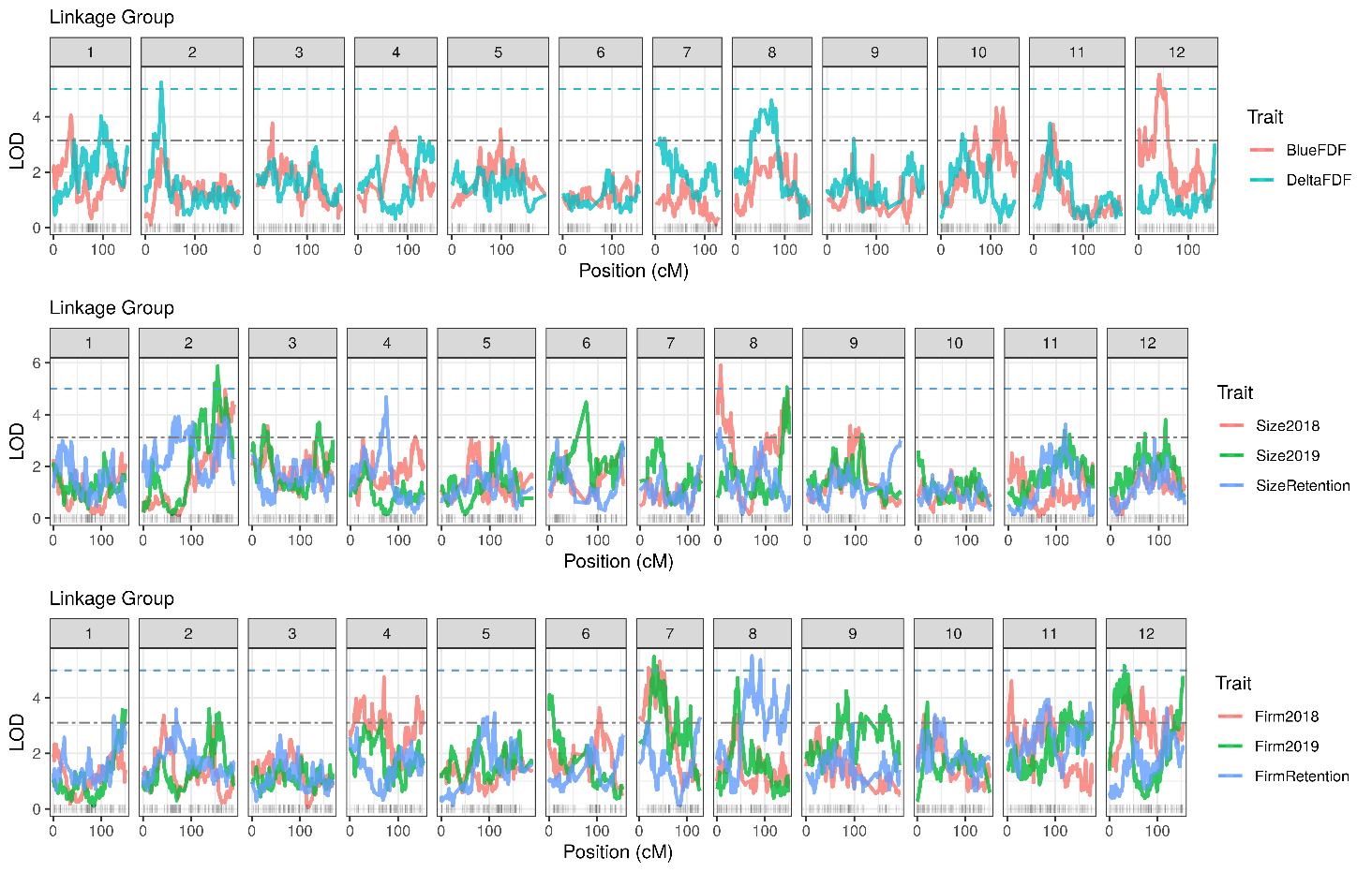


Supplementary Figure 2: QTL profiles for blueberry machine harvesting traits throughout the 12 linkage groups considering fixed effects interval mapping. LOD is the negative logarithm of the odds between a model with the QTL and a model without QTL. Blue dashed horizontal line is the threshold considering an alpha equal to 0.05 with Bonferroni correction. Black dashed line is the permutation threshold considering 95^o^ quantile of the second highest peak. Firmness retention, Blue FDF, and Delta FDF where measured only in year 2019.

## Supplementary Figure 3


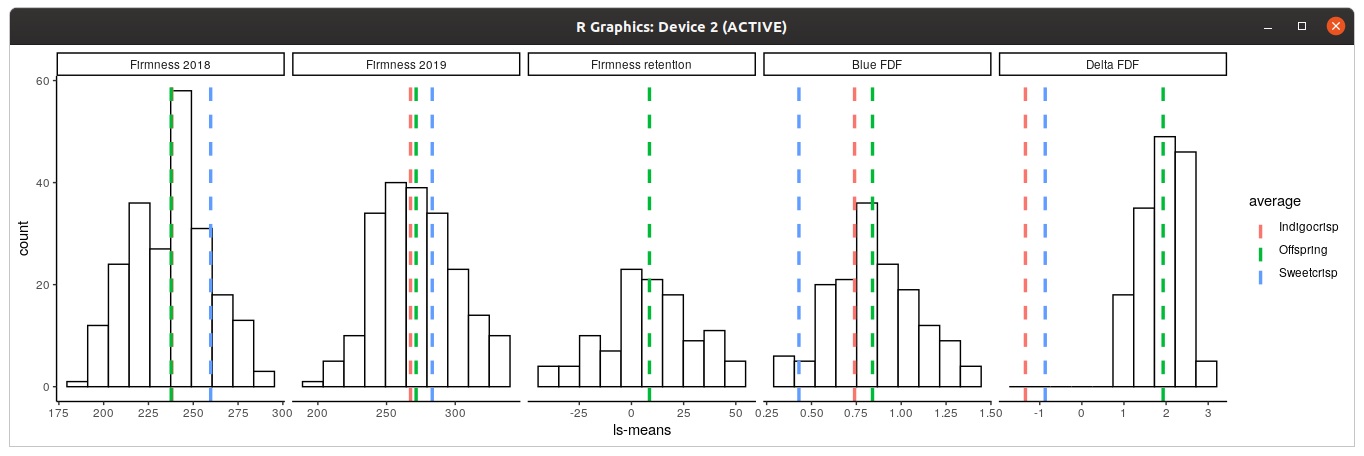


Supplementary Figure 3: Histograms of the ls-means for each trait. Vertical dashed lines represent the average ls-means for the parents (‘Indigocrisp’ and ‘Sweetcrisp’) and offspring.

## Supplementary Table 1

Attached SupTable1_Map.xlsx file with map information

The file Supplementary Table 1 contains 11 columns by 11,292 rows, where rows are the markers and columns present the assembled linkage group (LG), SNP name (scaffold + position), position in cM, parent 1 phase (a, b, c, d homologues), parent 2 phase (e, f, g, h). “|” represents the alternative SNP allele, and “o” otherwise (reference allele).

## Supplementary Table 2

Attached SuptTable2_Genes.xlsx file

The file Supplementary Table 2 contains four spreadsheets, one per trait, where rows are the predicted genes in QTL intervals, and columns have *in silico* annotation information: transcript ID, blastp description, protein length, e-value, mean percentage of identity, GO terms, GO description, enzyme codes, enzyme names, InterPro domains, and manual annotations, respectively.
